# A new species of *Cyrtodactylus* Gray, 1827 (Squamata, Gekkonidae) from Yunnan, China

**DOI:** 10.3897/zookeys.1021.60402

**Published:** 2021-03-02

**Authors:** Shuo Liu, Dingqi Rao

**Affiliations:** 1 Kunming Natural History Museum of Zoology, Kunming Institute of Zoology, Chinese Academy of Sciences, 32 Jiaochang Donglu, Kunming, Yunnan 650223, China Kunming Institute of Zoology, Chinese Academy of Sciences Kunming China; 2 Kunming Institute of Zoology, Chinese Academy of Sciences, 32 Jiaochang Donglu, Kunming, Yunnan 650223, China Kunming Institute of Zoology, Chinese Academy of Sciences Kunming China

**Keywords:** Bent-toed gecko, *Cyrtodactylus wayakonei*, karst-dwelling, taxonomy, Zhenkang

## Abstract

A new species of *Cyrtodactylus* is described on the basis of five specimens collected from the karst formations of Zhenkang County, Yunnan Province, China. *Cyrtodactylus zhenkangensis***sp. nov.** is recognized by having a unique combination of morphological characters, the most diagnostic being: 12–15 enlarged femoral scales on each thigh; 2–5 femoral pores on each thigh in males, 0–3 pitted scales on each thigh in females; eight or nine precloacal pores in a continuous row or separated by one poreless scale in males, 7–9 pitted scales in females; subcaudals enlarged, arranged alternately as single and double on anterior and mostly single at middle and posterior; dorsal surface of head with obvious reticulations. Phylogenetic analyses show that the new species is a member of the *C. wayakonei* species group and a sister taxon to a clade consisting of *C. wayakonei* and *C. martini* based on Maximum Likelihood analyses and Bayesian Inference and differs from its congeners by at least 12.0% genetic divergence in a fragment of the COI gene.

## Introduction

Bent-toed geckos of the genus *Cyrtodactylus* are one of the most species-diverse genera of gekkonid lizards (Kluge 2001; Uetz 2020), and many of these species are thought to be highly localized with extremely narrow geographic ranges (Nazarov et al. 2012; Luu et al. 2016; Grismer et al. 2018, 2020; Murdoch et al. 2019). At present, the genus contains more than 300 recognized species (Uetz et al. 2020), and approximately 150 new species have been described since 2010 and most of these new discoveries were from Southeast Asia (Schneider et al. 2020).

During our recent fieldwork in Yunnan Province, China, a series of bent-toed geckos was collected from the karst formations of Zhenkang County. Morphological and molecular phylogenetic analyses revealed that the new collection belonged to an unnamed species of *Cyrtodactylus*. We describe it as a new species.

## Materials and methods

### Sampling

Fieldwork was conducted at night. Specimens were collected by hand. Photographs were taken to document color pattern in life prior to euthanization. Liver tissues were stored in 99% ethanol and specimens were preserved in 75% ethanol. Specimens were deposited at Kunming Natural History Museum of Zoology, Kunming Institute of Zoology, Chinese Academy of Sciences (**KIZ**).

### Molecular analyses

Molecular data were generated for three specimens and analyzed with the available homologous sequences of the *Cyrtodactylus wayakonei* species group obtained from GenBank. The new sequences were deposited in GenBank under accession numbers MW593136–MW593138. Sequences of C. cf. interdigitalis Ulber, 1993 and *C. elok* Dring, 1979 were used as outgroups according to Nguyen et al. (2017) and Schneider et al. (2020).

We used the protocols of Le et al. (2006) for DNA extraction, amplification, and sequencing. DNA extraction used the standard three-step phenol/trichloromethane protocol (Sambrook et al. 1989). A fragment of the mitochondrial gene, cytochrome c oxidase subunit 1 (COI) was amplified in a volume consisted of 25 μl (10 μl of mastermix, 5 μl of water, 2 μl of each primer at 10 pmol/μl and 6 μl of DNA) by the polymerase chain reaction (PCR; 35 cycles of 95 °C for 30 s, 53 °C for 40 s, 72 °C for 90 s) and sequenced using the primer pair VF1-d (TTCTCAACCAACCACAARGAYATYGG) and VR1-d (TAGACTTCTGGGTGGCCRAARAAYCA) (Ivanova et al. 2006). PCR products were cleaned using ExoSAP-IT (Applied Biosystems) and sequenced in both directions by direct double strand cycle sequencing using the BigDye Terminator v. 3.1 Cycle Sequencing Kit on a 3130 DNA Analyzer (Applied Biosystems). Sequences were edited with Sequencher v. 5.4.6 (Gene Codes).

Sequences were aligned using ClustalW (Thompson et al. 1994) integrated in MEGA v. 7 (Kumar et al. 2016) with default parameters. Pairwise distances between species were calculated in MEGA v. 7 with the parameters Transitions + Transversions, Uniform rates, and Pairwise deletion (Kumar et al. 2016). The substitution model GTR+G+I was selected using the corrected Akaike Information Criterion (AICc) in MODELTEST v. 3.7 (Posada and Crandall 1998). Bayesian inference (BI) was performed in MrBayes v. 3.2.6 (Ronquist et al. 2012) based on the selected substitution model. Two runs were performed simultaneously with four Markov chains starting from random tree. The chains were run for 10,000,000 generations and sampled every 1000 generations. The first 25% of the sampled trees was discarded as burn-in after the standard deviation of split frequencies of the two runs reached a value of less than 0.01, and then the remaining trees were used to create a 50% majority-rule consensus tree and to estimate Bayesian posterior probabilities (BPP). Nodes with BPP of 95 and above were considered strongly supported (Huelsenbeck et al. 2001; Wilcox et al. 2002; Alfaro et al. 2003) and nodes with values of 90–94 as well supported (Chomdej et al. 2020). Maximum Likelihood (ML) analysis was performed in RaxmlGUI v. 1.5 (Silvestro and Michalak 2012), and nodal support was estimated by 1,000 rapid bootstrap replicates. Nodes with bootstrap values of 70 and above were considered significantly supported (Alfaro et al. 2003; Sitnikova 1996).

### Morphological analyses

Measurements were taken with digital calipers to the nearest 0.1 mm. Bilateral scale counts were given as left/right. The methodology of measurements and meristic counts followed Ngo (2011) and Schneider et al. (2020):

**AG** axilla to groin distance;

**DTR** dorsal tubercle rows, number of dorsal, longitudinal rows of tubercles at midbody between the ventrolateral folds;

**ED** ear diameter, greatest diameter of ear;

**EE** eye orbit to ear distance, from posterior corner of eye orbit to anterior margin of ear opening;

**EFS** enlarged femoral scales, number of enlarged femoral scale beneath each thigh;

**ForeaL** forearm length, from the base of the palm to the elbow;

**FP** femoral pores;

**GSDT** granular scales surrounding dorsal midbody tubercles;

**HH** maximum head height, from occiput to underside of jaws;

**HL** head length, from tip of snout to posterior margin of ear;

**HW** maximum head width;

**I** postrostrals or internasals;

**IFL** infralabials;

**IND** internarial distance, measured between inner borders of nostrils;

**IOD** interorbital distance, measured across narrowest point of frontal bone;

**LD4** subdigital lamellae under the fourth finger;

**LT4** subdigital lamellae under the fourth toe;

**ML** mental length;

**MW** mental width;

**OD** greatest diameter of orbit;

**PAT** postcloacal tubercles, number of tubercles on each side of postcloacal region;

**PM** postmentals, i.e. scales bordering mental shield, except infralabials;

**PP** precloacal pores;

**PVT** paravertebral tubercles, counted in a single paravertebral row from the level of the forelimb insertions to the level of the hind limb insertion;

**RH** rostral heigth;

**RW** rostral width;

**SC5SPL** scale rows between fifth supralabials;

**SE** snout to eye distance, from tip of snout to anterior corner of eye orbit;

**SL** shank length, from the base of heel to the knee;

**SPL** supralabials;

**SVL** snout-vent length, from tip of snout to anterior margin of cloaca;

**TaL** tail length, from posterior margin of cloaca to tip of tail;

**V** longitudinal ventral scale rows, counted across the belly between the ventrolateral folds at midbody.

Morphological comparisons and analyses were based on specimen examination and data obtained from the literature (Hoang et al. 2007; Rösler et al. 2008; Bauer et al. 2009, 2010; Ngo and Grismer 2010; Nguyen et al. 2010, 2015, 2017; Sumontha et al. 2010; Teyníe and David 2010; Luu et al. 2011, 2013, 2016; Ngo 2011; Ngo and Chan 2011; Schneider et al. 2011, 2014, 2020; Kunya et al. 2014; Nazarov et al. 2014, 2018; Nguyen et al. 2014; Le 2016; Pham et al. 2019).

## Results

### Molecular analyses

**Figure 1.**
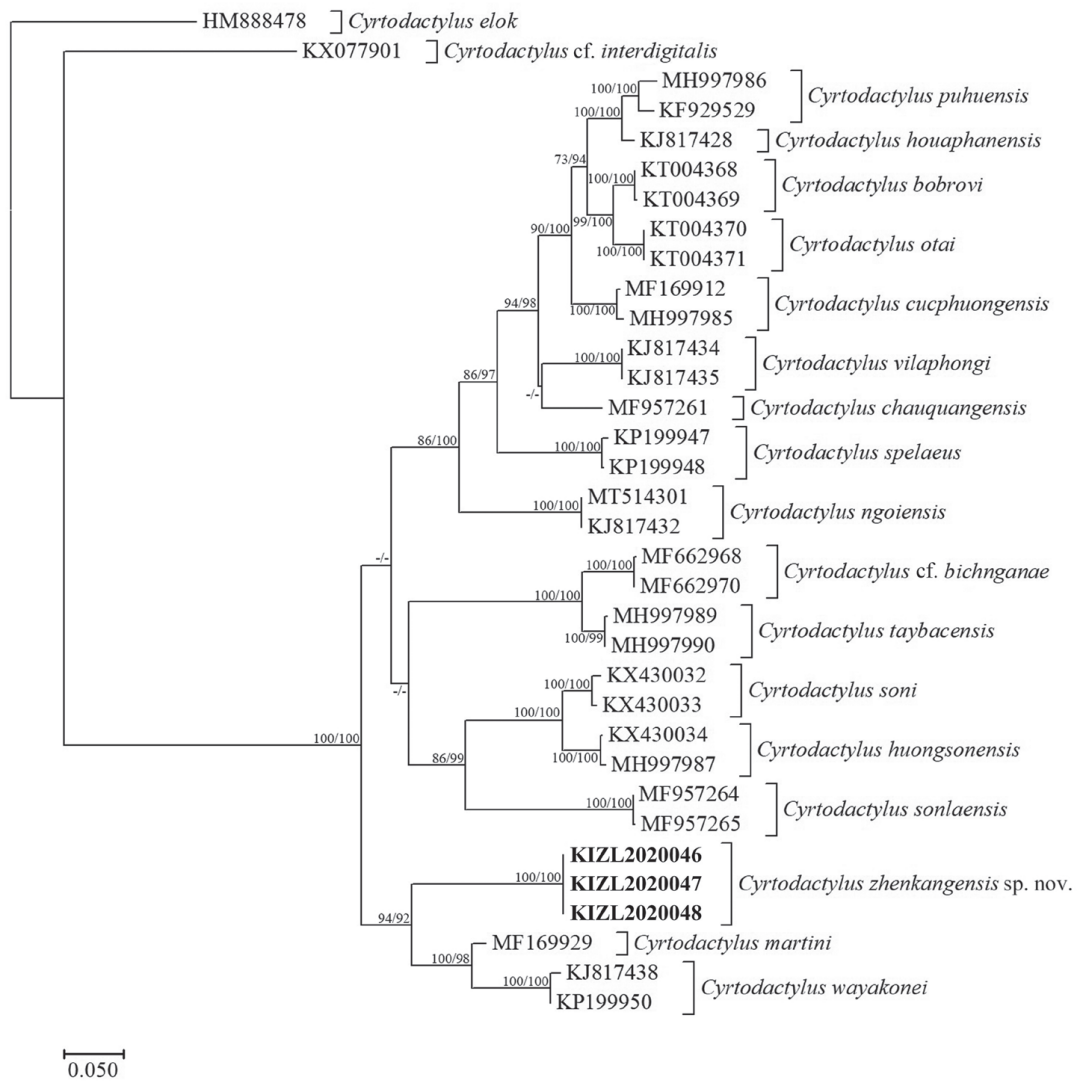
Bayesian Inference phylogram inferred from partial COI genes. Numbers before slashes indicate bootstrap support for Maximum Likelihood analyses and numbers after slashes indicate Bayesian posterior probabilities. The symbol “–” represents the value below 60.

The obtained sequence alignment is 690 bp in length. The topologies derived from ML and BI analyses were similar and basically consistent with those of Nguyen et al. (2017), Pham et al. (2019), and Schneider et al. (2020). The sequences of three specimens collected from Zhenkang County, Yunnan, China were nested them within the *Cyrtodactylus wayakonei* group and the sister group to a clade consisting of *C. wayakonei* Nguyen, Kingsada, Rösler, Auer & Ziegler, 2010 and *C. martini* Ngo, 2011 with strong support in ML and moderate support in BI (Fig. 1). The interspeciﬁc uncorrected genetic *p*-distances between the newly collected specimens and other members of *C. wayakonei* group ranged from 12.0% to 17.8% (Table 1).

**Table 1.** Mean uncorrected pairwise genetic distances (%) based on 690 base pairs of COI gene sequences.

		1	2	3	4	5	6	7	8	9	10	11	12	13	14	15	16	17	18
**1**	*Cyrtodactylus bichnganae*																		
**2**	*C. bobrovi*	17.2																	
**3**	*C. chauquangensis*	15.3	9.3																
**4**	*C. cucphuongensis*	16.6	6.9	8.0															
**5**	*C. houaphanensis*	17.3	6.2	8.8	7.2														
**6**	*C. huongsonensis*	14.7	15.8	14.5	14.9	16.0													
**7**	*C. martini*	15.6	15.1	13.7	14.1	15.6	15.0												
**8**	*C. ngoiensis*	15.9	12.3	11.6	13.8	13.1	14.1	14.7											
**9**	*C. otai*	16.7	3.8	9.3	7.2	6.2	15.4	15.9	13.1										
**10**	*C. puhuensis*	18.8	7.2	10.3	8.2	3.4	17.7	16.1	15.0	7.3									
**11**	*C. soni*	13.6	16.7	14.5	15.8	16.4	5.3	15.4	15.0	16.3	18.7								
**12**	*C. sonlaensis*	15.4	16.3	16.8	16.7	17.7	13.4	14.6	15.4	17.3	18.8	14.0							
**13**	*C. spelaeus*	16.9	10.0	11.7	12.1	10.9	16.7	15.0	13.5	11.2	11.8	15.8	15.7						
**14**	*C. taybacensis*	5.2	16.1	14.4	15.4	16.2	15.1	14.7	15.6	16.4	17.5	14.4	16.1	15.3					
**15**	*C. vilaphongi*	16.4	9.3	8.2	9.5	8.2	15.3	14.7	12.9	9.4	10.1	15.9	17.1	11.8	15.9				
**16**	*C. wayakonei*	15.2	16.7	15.2	16.5	17.4	16.7	6.5	15.5	18.3	18.1	17.5	15.9	16.2	15.6	16.0			
**17**	*Cyrtodactylus zhenkangensis* sp. nov.	**17.8**	**15.0**	**14.9**	**15.8**	**16.2**	**16.7**	**12.0**	**14.3**	**16.1**	**16.8**	**17.1**	**17.1**	**15.7**	**16.8**	**16.4**	**13.1**		
**18**	*C. interdigitalis*	18.5	19.9	19.2	19.3	20.2	20.2	18.2	20.1	20.1	21.9	19.9	20.5	20.4	18.3	19.3	18.3	**19.9**	
**19**	*C. elok*	18.8	19.4	18.9	17.9	19.5	19.6	17.1	19.5	19.6	20.2	20.2	19.7	18.6	18.6	19.2	18.6	**19.5**	15.8

### Taxonomic accounts

#### Cyrtodactylus zhenkangensis sp. nov.

Taxon classificationAnimaliaSquamataGekkonidae

66B54C01-4996-559C-81B8-86F13C2AF3B1

http://zoobank.org/1CAE09BE-E522-42EF-AD5A-0E3B7A694CDB

Figs 2 , 3 , 4 , 5

##### Holotype.

KIZL2020049, adult male, China, Yunnan Province, Lincang City, Zhenkang County, Nansan town, 23°46'32"N, 98°50'28"E, 1060 m elevation, collected on 11 September 2020 by Shuo Liu.

##### Paratypes.

KIZL2020048 and KIZL2020050, two adult females; KIZL2020046, subadult male; and KIZL2020047, subadult female; all the same collection data as the holotype.

##### Etymology.

The name refers to Zhenkang County, where the new species was found.

##### Diagnosis.

*Cyrtodactylus zhenkangensis* sp. nov. differs from all other congeners by the following combination of characters: medium size (SVL 78.1–87.4 mm); ventrolateral folds present with interspersed tubercles; 12–15 enlarged femoral scales on each thigh; 2–5 femoral pores on each thigh in males, 0–3 pitted scales on each thigh in females; eight or nine precloacal pores in a continuous row or separated by one poreless scale in males, 7–9 pitted scales in females; two or three postcloacal tubercles on each side; 18–21 lamellae under finger IV, 21–23 lamellae under toe IV; subcaudals enlarged, arranged alternately as single and double on anterior and mostly single at middle and posterior; dorsal surface of head with obvious, light-colored reticulations; eight or nine irregular transverse bands on the dorsum of body.

##### Description of holotype.

Adult male, SVL 87.4 mm; head distinguished from neck, moderately long (HL/SVL 0.27), relatively widened (HW/HL 0.79), slightly depressed (HH/HL 0.48); two supranasals separated by one internasal; nares oval, surrounded by supranasal, rostral, first supralabial, and three or four postnasals; loreal region concave; snout long (SE/HL 0.41), round anteriorly, longer than diameter of orbit (OD/SE 0.70); snout scales small, round, granular, larger than those in frontal and parietal regions; eye large (OD/HL 0.28), pupils vertical; upper eyelid fringe with spinous scales; ear opening oval, obliquely directed, small in size (ED/HL 0.08); rostral wider than high (RH/RW 0.66), medially divided dorsally by a suture, reaching to approximately half-way down rostral, in contact with first supralabial and nostrils laterally, and supranasals and internasal dorsally; mental triangular, narrower than rostral (MW/RW 0.83), wider than high (ML/MW 0.82); two postmentals, enlarged, in contact posteriorly, bordered by mental anteromedially, first infralabial anterolaterally, and an enlarged chin scale posterolaterally; 10/10 supralabials; 10/10 infralabials.

Body slender (AG/SVL 0.41), ventrolateral folds slightly developed with interspersed tubercles; dorsal scales granular; dorsal tubercles round and weakly keeled, four or five times larger than the size of adjoining scales, conical, present on occiput, back and tail base, each surrounded by nine or ten granular scales, in 24 irregular longitudinal rows at the midbody, 29 paravertebral tubercles; ventral scales smooth, larger than those of dorsum, round, subimbricate, largest posteriorly, in 33 longitudinal rows at midbody; gular region with homogenous smooth scales; precloacal groove absent; three rows of enlarged scales present in posterior region of pore-bearing scales; 13/15 enlarged femoral scales beneath thighs continuous with enlarged precloacal scales; femoral pores bearing scales separated from pore-bearing precloacal scales by six poreless or pitted femoral scales on the left side and nine poreless or pitted femoral scales on the right side; 5/5 femoral pores; 5/3 precloacal pores, separated by one poreless scale; most precloacal pores are positioned in the posterior margin of their scales and femoral pores positioned in the center of scales.

**Figure 2.**
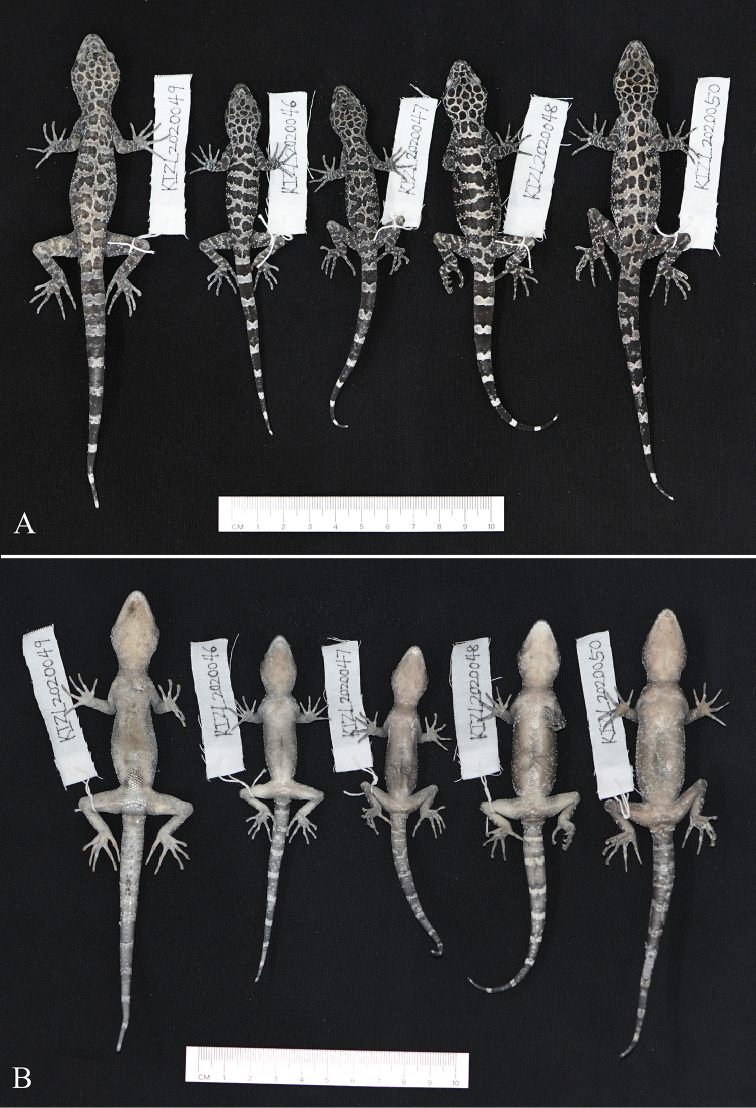
Type series of *Cyrtodactylus zhenkangensis* sp. nov. in preservative **A** dorsal view **B** ventral view.

Fore and hind limbs moderately slender (ForeaL/SVL 0.17, SL/SVL 0.20); dorsal surface of forelimbs covered by a few weakly developed tubercles; interdigital webbing absent; lamellae under finger IV 20/18, under toe IV 21/23; relative length of fingers I<II<V<III <IV, relative length of toes I<II<III<V<IV.

**Figure 3.**
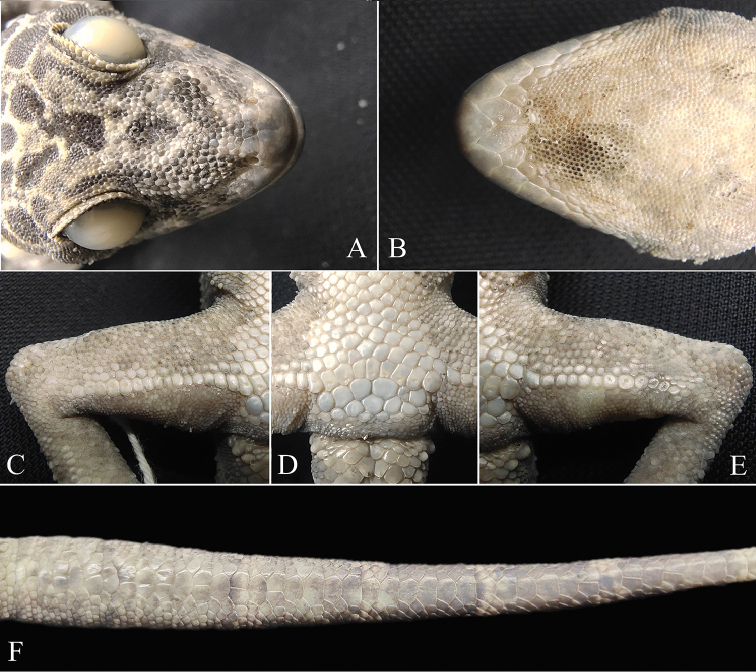
Close-up views of the holotype (KIZL20200049) of *Cyrtodactylus zhenkangensis* sp. nov. in preservative **A** dorsal view of head **B** ventral view of head **C** right side femoral region **D** precloacal region **E** left side femoral region **F** subcaudal scales.

Tail complete, longer than snout-vent length (TaL/SVL 1.12); 2/3 postcloacal tubercles; dorsal tail base with tubercles; subcaudals smooth, enlarged, arranged alternately in single and double series at anterior and mostly singly at middle and posterior parts.

***Color of holotype in life.*** Head brown with pale-yellow, slightly symmetrical reticulations on either side of the midline, no dark-colored nuchal loop; dorsum of body brown with approximately nine pale-yellow, transverse, irregular bands from forelimb insertions to base of tail and one longitudinal, continuous, narrow vertebral stripe; dorsal surface of limbs brown with some light-yellow, irregularly shaped bands, some small, light-yellow spots on the dorsum of fingers and toes; ventral surface of head, body, and limbs grey with no stripes or spots; tail brownish black with ten yellowish white rings; iris copper-yellow.

**Figure 4.**
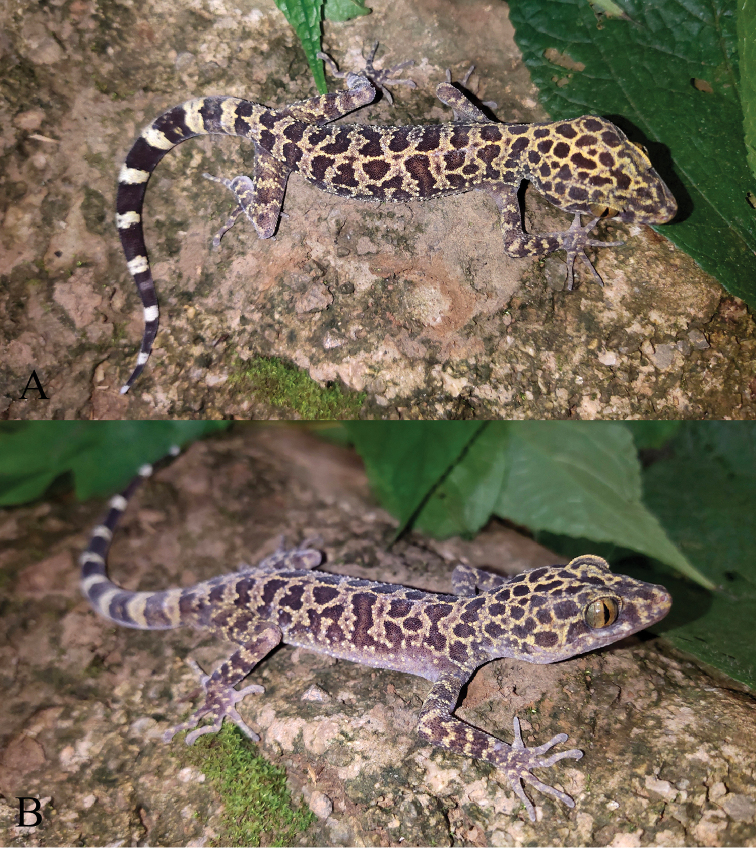
The holotype (KIZL20200049) of *Cyrtodactylus zhenkangensis* sp. nov. in life **A** dorsal view **B** lateral view.

**Figure 5.**
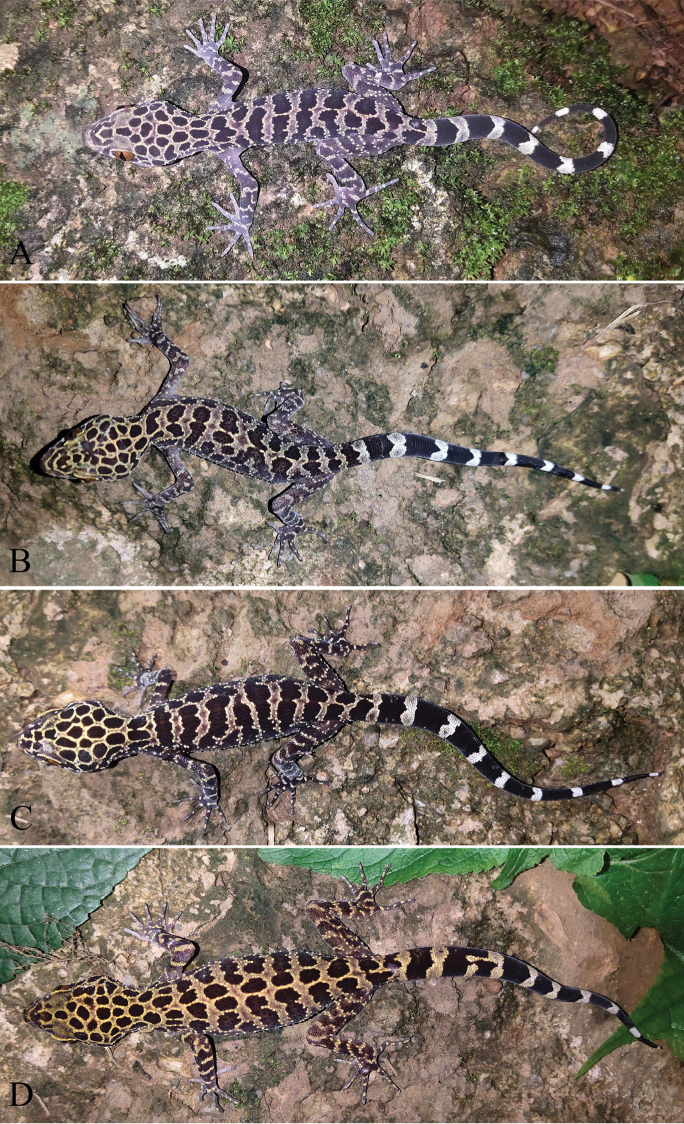
The paratypes of *Cyrtodactylus zhenkangensis* sp. nov. in life **A** subadult male (KIZL2020046) **B** subadult female (KIZL2020047) **C** adult female (KIZL2020048) **D** adult female (KIZL2020050).

***Variations.*** Color pattern variations are shown in Figure 5, and morphometric and meristic differences are presented in Table 2. Morphologically the paratypes resemble the holotype except as follows: KIZL2020046 and KIZL2020047 each has one vertebral stripe like the holotype but it is discontinuous; KIZL2020050 has one continuous vertebral strip and two discontinuous, longitudinal, narrow stripes on the sides of vertebral strip; KIZL2020048 only has transverse bands and no vertebra stripe. All paratypes have continuous precloacal pores (pitted) and fewer femoral pores (pitted).

**Table 2.** Measurements (mm) and meristic data for the type series of *Cyrtodactylus zhenkangensis* sp. nov. Abbreviations defined in Materials and methods.

	KIZL2020049 Holotype	KIZL2020046 Paratype	KIZL2020047 Paratype	KIZL2020048 Paratype	KIZL2020050 Paratype
Sex	Male	Subadult male	Subadult female	Female	Female
SVL	87.4	64.1	66.2	78.1	85.5
TaL	98.1	73.2	76.3	86.9	96.8
HH	11.5	9.0	8.5	10.3	10.4
HL	23.9	18.6	18.6	22.0	24.2
HW	18.8	13.9	14.2	17.1	17.8
OD	6.8	5.1	5.3	6.3	6.8
SE	9.7	7.9	8.0	9.3	9.9
EE	7.6	5.7	5.8	6.7	7.1
IND	3.1	2.5	2.6	3.0	3.2
IOD	8.3	5.8	6.1	7.2	7.7
ED	1.8	1.4	1.3	1.3	1.8
AG	35.5	25.7	25.4	33.2	36.1
ForeaL	15.2	11.5	11.6	13.1	14.5
SL	17.8	13.0	13.5	15.9	16.7
RW	4.1	3.3	3.2	3.7	4.2
RH	2.7	2.0	1.6	2.0	2.4
MW	3.4	3.1	2.7	3.2	3.8
ML	2.8	2.2	2.2	2.1	2.9
SPL	10/10	10/10	10/11	11/10	10/10
IFL	10/10	8/8	10/10	9/9	8/7
I	1	1	1	1	1
SC5SPL	37	32	34	28	33
PM	2	2	2	2	2
GSDT	9–10	9–10	8–9	8–10	8–9
DTR	24	23	21	20	22
PVT	29	27	32	33	28
V	33	32	32	34	33
EFS	13/15	14/14	14/13	13/12	14/15
PP	8	9 (pitted)	9 (pitted)	8 (pitted)	7 (pitted)
FP	5/5	2/2 (pitted)	1/0 (pitted)	3/0 (pitted)	2/2 (pitted)
PAT	2/3	2/2	2/2	3/3	2/3
LD4	20/18	19/20	19/18	21/20	21/19
LT4	21/23	23/22	22/22	22/21	22/22

##### Distribution.

The new species is currently known only from the type locality in Zhenkang County, Yunnan Province, China.

##### Natural history.

All specimens were found at night between 19:00 and 21:00 on limestone cliffs of the karst formations. The surrounding habitat was primary forestwith a stream nearby. No eggs or juveniles were found.

##### Comparisons.

*Cyrtodactylus zhenkangensis* sp. nov. is distinguishable from all other members of the *C. wayakonei* group by a unique combination of morphological characters. *Cyrtodactylus zhenkangensis* sp. nov. differs from *C. bichnganae* Ngo & Grismer, 2010; *C. huongsonensis* Luu, Nguyen, Do & Ziegler, 2011; and *C. sonlaensis* Nguyen, Pham, Ziegler, Ngo & Le, 2017 in having fewer femoral pores in males (4–10 vs 15–29).

*Cyrtodactylus zhenkangensis* sp. nov. differs from *C. bobrovi* Nguyen, Le, Pham, Ngo, Hoang, Pham & Ziegler, 2015; *C. otai* Nguyen, Le, Pham, Ngo, Hoang, Pham & Ziegler, 2015; and *C. vilaphongi* Schneider, Nguyen, Le, Nophaseud, Bonkowski & Ziegler, 2014 in having enlarged subcaudal scales (vs lacking enlarged subcaudals).

*Cyrtodactylus zhenkangensis* sp. nov. differs from *C. chauquangensis* Hoang, Orlov, Ananjeva, Johns, Hoang & Dau, 2007; *C. cucphuongensis* Ngo & Chan, 2011; *C. houaphanensis* Schneider, Luu, Sitthivong, Teynié, Le, Nguyen & Ziegler, 2020; *C. puhuensis* Nguyen, Yang, Le, Nguyen, Orlov, Hoang, Nguyen, Jin, Rao, Hoang, Che, Murphy & Zhang, 2014; *C. spelaeus* Nazarov, Poyarkov, Orlov, Nguyen, Milto, Martynov, Konstantinov & Chulisov, 2014; and *C. taybacensis* Pham, Le, Ngo, Ziegler & Nguyen, 2019 in having femoral pores in males (vs lacking femoral pores in males).

*Cyrtodactylus zhenkangensis* sp. nov. differs from *C. martini* in having femoral pores in males (vs lacking femoral pores in males) and more irregular transverse bands on the dorsum of body (8–9 vs 5–7).

*Cyrtodactylus zhenkangensis* sp. nov. differs from *C. ngoiensis* Schneider, Luu, Sitthivong, Teynié, Le, Nguyen & Ziegler, 2020 and *C. soni* Le, Nguyen, Le & Ziegler, 2016 in its smaller body size (64.1–87.4 mm vs 62.9–103 mm) and having more lamellae under finger IV (18–21 vs 15–19) and toe IV (21–23 vs 18–22).

**Figure 6.**
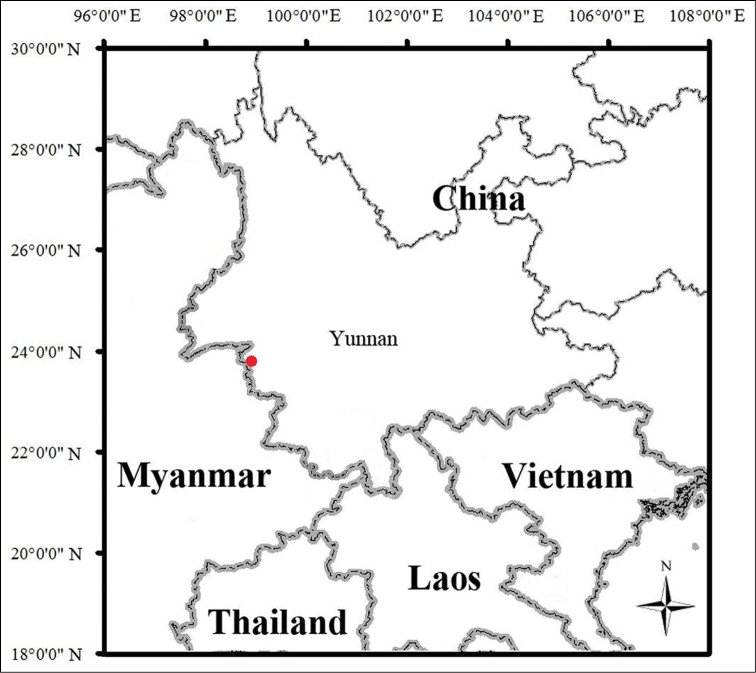
Map showing the type locality (red dot) of *Cyrtodactylus zhenkangensis* sp. nov. in Zhenkang County, Yunnan Province, China.

*Cyrtodactylus zhenkangensis* sp. nov. differs from *C. wayakonei* in having enlarged subcaudal scales (vs lacking enlarged subcaudals) and with more irregular transverse bands on the dorsum of body (8–9 vs 5–7).

For other species which were not included in the phylogenetic analyses and resemble *Cyrtodactylus zhenkangensis* sp. nov. in morphology. *Cyrtodactylus zhenkangensis* sp. nov. differs from *C. auribalteatus* Sumontha, Panitvong & Deein, 2010 in having more transverse bands on the dorsum of body (8–9 vs 4–5), obvious reticulations on the dorsum of head (vs no obvious reticulations) and absent dark-colored nuchal loop (vs present).

*Cyrtodactylus zhenkangensis* sp. nov. differs from *C. doisuthep* Kunya, Panmongkol, Pauwels, Sumontha, Meewasana, Bunkhwamdi & Dangsri, 2014 in having fewer femoral pores (0–10 vs 12–14), more precloacal pores (7–9 vs 6), and absent dark-colored nuchal loop (vs present).

*Cyrtodactylus zhenkangensis* sp. nov. differs from *C. dumnuii* Bauer, Kunya, Sumontha, Niyomwan, Pauwels, Chanhome & Kunya, 2010 in having more lamellae under toe IV (21–23 vs 19), absent dark-colored nuchal loop (vs present), and obvious reticulations on the dorsum of head (vs not obvious or no reticulations).

*Cyrtodactylus zhenkangensis* sp. nov. differs from *C. erythrops* Bauer, Kunya, Sumontha, Niyomwan, Panitvong, Pauwels, Chanhome & Kunya, 2009 in having fewer femoral pores in males (4–10 vs 18–20), more lamellae under finger IV (18–21 vs 16) and toe IV (21–23 vs 20), and more transverse bands on the dorsum of body (8–9 vs 6–7).

**Figure 7.**
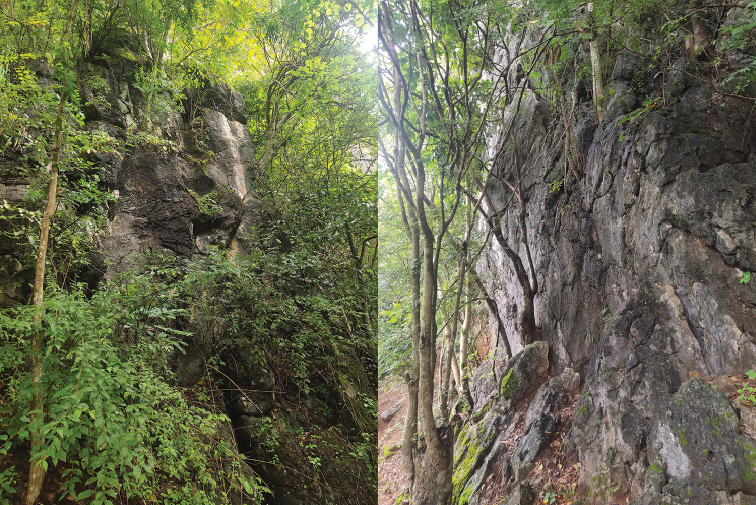
Habitat of *Cyrtodactylus zhenkangensis* sp. nov. at the type locality in Zhenkang County, Yunnan Province, China.

## Discussion

According to Pham et al. (2019) and Schneider et al. (2020), the *Cyrtodactylus wayakonei* species group contains 16 species, namely *C. bichnganae*, *C. bobrovi*, *C. chauquangensis*, *C. cucphuongensis*, *C. houaphanensis*, *C. huongsonensis*, *C. martini*, *C. ngoiensis*, *C. otai*, *C. puhuensis*, *C. soni*, *C. sonlaensis*, *C. spelaeus*, *C. taybacensis*, *C. vilaphongi*, and *C. wayakonei*. However, we speculate that there are still some other species (e.g., *C. auribalteatus*, *C. doisuthep*, *C. dumnuii*, and *C. erythrops*) which were not included in the phylogenetic analyses also belong to this species group based on morphology, molecular evidence is needed to clarify these problems.

Although the distribution of the new species is distant from the distributions of *C. martini* and *C. wayakonei*, the new species is most similar to the latter two in both morphology and phylogeny. The new species is not found in a protected area; the type locality is just beside the county seat, where there are human activities during the day but usually not at night. This species is nocturnal, so it may be less affected by human activities.

There are many other karst formations in Yunnan, some of which remain insufficiently surveyed. We are continuing to conduct more expeditions in these regions, and it is likely that additional new species of *Cyrtodactylus* will be found in these karst systems.

## Supplementary Material

XML Treatment for Cyrtodactylus zhenkangensis

## References

[B1] Alfaro ME Zoller S Lutzoni F (2003) Bayes or bootstrap? A simulation study comparing the performance of Bayesian Markov chain Monte Carlo sampling and bootstrapping in assessing phylogenetic confidence. Molecular Biology and Evolution 20: 255–266. 10.1093/molbev/msg028 12598693

[B2] Bauer AM Kunya K Sumontha M Niyomwan P Panitvong N Pauwels OSG Chanhome L Kunya T (2009) *Cyrtodactylus erythrops* (Squamata: Gekkonidae), a new cave-dwelling gecko from Mae Hong Son Province, Thailand. Zootaxa 3811: 251–261. 10.11646/zootaxa.2124.1.4

[B3] Bauer A Kunya K Sumontha M Niyomwan P Pauwels OSG Chanhome L Kunya T (2010) *Cyrtodactylus dumnuii* (Squamata: Gekkonidae), a new cave-dwelling gecko from Chiang Mai Province, Thailand. Zootaxa 2570: 41–50. 10.11646/zootaxa.2570.1.2

[B4] Chomdej S Suwannapoom C Pawangkhanant P Pradit W Nazarov RA Grismer LL Poyarkov NA (2020) A new species *Cyrtodactytlus* Gray (Squamata: Gekkonidae) from western Thailand and the phylogenetic placement of *C. inthanon* and *C. doisuthep*. Zootaxa 4838: 179–209. 10.11646/zootaxa.4838.2.2 33056821

[B5] Dring JCM (1979) Amphibians and reptiles from northern Trengganu, Malaysia, with descriptions of two new geckos: *Cnemaspis* and *Cyrtodactylus*. Bulletin of the British Museum (Natural History), Zoology 34: 181–241.

[B6] Grismer LL Wood Jr PL Thura MK Zin T Quah ESH Murdoch ML Grismer MS Lin A Kyaw H Ngwe L (2018) Twelve new species of *Cyrtodactylus* Gray (Squamata: Gekkonidae) from isolated limestone habitats in east-central and southern Myanmar demonstrate high localized diversity and unprecedented microendemism. Zoological Journal of the Linnean Society 182: 862–959. 10.1093/zoolinnean/zlx057

[B7] Grismer LL Wood Jr PL Le MD Quah ESH Grismer JL (2020) Evolution of habitat preference in 243 species of bent-toed geckos (genus *Cyrtodactylus* Gray, 1827) with a discussion of karst habitat conservation. Ecology and Evolution 10: 13717–13730. 10.1002/ece3.6961 33391675 PMC7771171

[B8] Hoang QX Orlov NL Ananjeva NB Johns AG Hoang TN Dau VQ (2007) Description of a new species of the genus *Cyrtodactylus* Gray, 1827 (Squamata: Sauria: Gekkonidae) from the karst of North Central Vietnam. Russian Journal of Herpetology 14: 98–106.

[B9] Ivanova NV Dewaard JR Hebert PDN (2006) An inexpensive, automation-friendly protocol for recovering high-quality DNA. Molecular Ecology Notes 6: 998–1002. 10.1111/j.1471-8286.2006.01428.x

[B10] Kluge AG (2001) Gekkotan lizard taxonomy. Hamadryad 26: 1–209.

[B11] Kumar S Stecher G Tamura K (2016) MEGA7: Molecular Evolutionary Genetics Analysis version 7.0 for bigger datasets. Molecular Biology and Evolution 33: 1870–1874. 10.1093/molbev/msw054 27004904 PMC8210823

[B12] Kunya K Panmongkol A Pauwels OGS Sumontha M Meewasana J Bunkhwamdi W Dangsri S (2014) A new forest-dwelling Bent-toed Gecko (Squamata: Gekkonidae: *Cyrtodactylus*) from Doi Suthep, Chiang Mai Province, northern Thailand. Zootaxa 3811: 251–261. 10.11646/zootaxa.3811.2.6 24943162

[B13] Huelsenbeck JP Ronquist F Nielsen R Bollback JP (2001) Bayesian Inference of phylogeny and its impact on evolutionary biology. Science 294: 2310–2314. 10.1126/science.1065889 11743192

[B14] Le DT Nguyen TQ Le MD Ziegler T (2016) A new species of *Cyrtodactylus* (Squamata: Gekkonidae) from Ninh Binh Province, Vietnam. Zootaxa 4162: 268–282. 10.11646/zootaxa.4162.2.4 27615973

[B15] Le M Raxworthy CJ McCord WP Mertz L (2006) A molecular phylogeny of tortoises (Testudines: Testudinidae) based on mitochondrial and nuclear genes. Molecular Phylogenetics and Evolution 40: 517–531. 10.1016/j.ympev.2006.03.003 16678445

[B16] Luu VQ Bonkowski M Nguyen TQ Le MD Schneider N Ngo HT Ziegler T (2016) Evolution in karst massifs: cryptic diversity among bent-toed geckos along the Truong Son Range with descriptions of three new species and one new country record from Laos. Zootaxa 4107: 101–140. 10.11646/zootaxa.4107.2.1 27394811

[B17] Luu VQ Calame T Nguyen TQ Soudthichak S Bonkowski M Ziegler T (2013) New country records of reptiles from Laos. Biodiversity Data Journal 1: e1015. 10.3897/BDJ.1.e1015 PMC3964705 24723754

[B18] Luu VQ Nguyen TQ Do HQ Ziegler T (2011) A new *Cyrtodactylus* (Squamata: Gekkonidae) from Huong Son limestone forest, Hanoi, northern Vietnam. Zootaxa 3129: 39–50. 10.11646/zootaxa.3129.1.3

[B19] Murdoch ML Grismer LL Wood Jr PL Neang T Poyarkov NA Ngo TV Nazarov RA Aowphol A Pauwels OSG Nguyen HN Grismer JL (2019) Six new species of the *Cyrtodactylus intermedius* complex (Squamata: Gekkonidae) from the Cardamom Mountains and associated highlands of Southeast Asia. Zootaxa 4554: 1–62. 10.11646/zootaxa.4554.1.1 30790979

[B20] Nazarov RA Pauwels OSG Konstantinov EL Chulisov AS Orlov NL Poyarkov NA (2018) A new karst-dwelling bent-toed gecko (Squamata: Gekkonidae: *Cyrtodactylus*) from Xiangkhoang Province, northeastern Laos. Zoological Research 39: 197–213. 10.24272/j.issn.2095-8137.2018.010 PMC5968862 29683111

[B21] Nazarov RA Poyarkov NA Orlov NL Nguyen SN Milto KD Martynov AA Konstantinov EL Chulisov AS (2014) A review of genus *Cyrtodactylus* (Reptilia: Sauria: Gekkonidae) in fauna of Laos with description of four new species. Proceedings of the Zoological Institute RAS 318: 391–423.

[B22] Nazarov RA Poyarkov NA Orlov NL Phung TM Nguyen TT Hoang DM Ziegler T (2012) Two new cryptic species of the *Cyrtodactylus irregularis* complex (Squamata: Gekkonidae) from southern Vietnam. Zootaxa 3302: 1–24. 10.11646/zootaxa.3302.1.1

[B23] Ngo TV (2011) *Cyrtodactylus martini*, another new karst-dwelling *Cyrtodactylus* Gray, 1827 (Squamata: Gekkonidae) from Northwestern Vietnam. Zootaxa 2834: 33–46. 10.11646/zootaxa.2834.1.3

[B24] Ngo TV Chan KO (2011) A new karstic cave-dwelling *Cyrtodactylus* Gray (Squamata: Gekkonidae) from Northern Vietnam. Zootaxa 3125: 51–63.

[B25] Ngo TV Grismer LL (2010) A new karst dwelling *Cyrtodactylus* (Squamata: Gekkonidae) from Son La Province, northwestern Vietnam. Hamadryad 35: 84–95. 10.11646/zootaxa.2652.1.1

[B26] Nguyen TQ Kingsada P Rösler H Auer M Ziegler T (2010) A new species of *Cyrtodactylus* (Squamata: Gekkonidae) from northern Laos. Zootaxa 2652: 1–16.

[B27] Nguyen TQ Le MD Pham AV Ngo HN Hoang CV Pham CT Ziegler T (2015) Two new species of *Cyrtodactylus* (Squamata: Gekkonidae) from the karst forest of Hoa Binh Province, Vietnam. Zootaxa 3985: 375–390. 10.11646/zootaxa.3985.3.3 26250040

[B28] Nguyen TQ Pham AV Ziegler T Ngo HT Le MD (2017) A new species of *Cyrtodactylus* (Squamata: Gekkonidae) and the first record of *C. otai* from Son La Province, Vietnam. Zootaxa 4341: 25–40. 10.11646/zootaxa.4341.1.2 29245698

[B29] Nguyen SN Yang JX Le NT Nguyen LT Orlov NL Hoang CV Nguyen TQ Jin JQ Rao DQ Hoang TN Che J Murphy RW Zhang YP (2014) DNA barcoding of Vietnamese bent-toed geckos (Squamata: Gekkonidae: *Cyrtodactylus*) and the description of a new species. Zootaxa 3784: 48–66. 10.11646/zootaxa.3784.1.2 24872031

[B30] Pham AV Le MD Ziegler T Nguyen TQ (2019) A new species of *Cyrtodactylus* (Squamata: Gekkonidae) from northwestern Vietnam. Zootaxa 4544: 360–380. 10.11646/zootaxa.4544.3.3 30647245

[B31] Posada D Crandall KA (1998) Modeltest: testing the model of DNA substitution. Bioinformatics 14: 817–818. 10.1093/bioinformatics/14.9.817 9918953

[B32] Ronquist F Teslenko M van der Mark P Ayres DL Darling A Höhna S Larget B Liu L Suchard MA Huelsenbeck JP (2012) MrBayes 3.2: efficient Bayesian phylogenetic inference and model choice across a large model space. Systematic Biology 61: 539–542. 10.1093/sysbio/sys029 22357727 PMC3329765

[B33] Rösler H Vu TN Nguyen TQ Ngo TV Ziegler T (2008) A new *Cyrtodactylus* (Squamata: Gekkonidae) from central Vietnam. Hamadryad 33: 48–63.

[B34] Schneider N Luu VQ Sitthivong S Teynié A Le MD Nguyen TQ Ziegler T (2020) Two new species of *Cyrtodactylus* (Squamata: Gekkonidae) from northern Laos, including new finding and expanded diagnosis of *C. bansocensis*. Zootaxa 4822: 503–530. 10.11646/zootaxa.4822.4.3 33056268

[B35] Schneider N Nguyen TQ Le MD Nophaseud L Bonkowski M Ziegler T (2014) A new species of *Cyrtodactylus* (Squamata: Gekkonidae) from the karst forest of northern Laos. Zootaxa 3835: 80–96. 10.11646/zootaxa.3835.1.4 25081436

[B36] Schneider N Nguyen TQ Schmitz A Kingsada P Auer M Ziegler T (2011) A new species of karst dwelling *Cyrtodactylus* (Squamata: Gekkonidae) from northwestern Laos. Zootaxa 2930: 1–21. 10.11646/zootaxa.2930.1.1

[B37] Silvestro D Michalak I (2012) raxmlGUI: a graphical front-end for RAxML. Organisms Diversity and Evolution 12: 335–337. 10.1007/s13127-011-0056-0

[B38] Sitnikova T (1996) Bootstrap method of interior-branch test for phylogenetic trees. Molecular Biology and Evolution 13: 605–611. 10.1093/oxfordjournals.molbev.a025620 8882503

[B39] Sumontha M Panitvong N Deein G (2010) *Cyrtodactylus auribalteatus* (Squamata: Gekkonidae), a new cave-dwelling gecko from Phitsanulok Province, Thailand. Zootaxa 2370: 53–64. 10.11646/zootaxa.2370.1.3

[B40] Teyníe A David P (2010) Voyages naturalists au Laos. Les reptiles. Revoir Editions, Chamalières, France, 315 pp.

[B41] Thompson JD Higgins DG Gibson TJ (1994) CLUSTAL W: improving the sensitivity of progressive multiple sequence alignment through sequence weighting, position-specific gap penalties and weight matrix choice. Nucleic Acids Research 22: 4673–4680. 10.1093/nar/22.22.4673 7984417 PMC308517

[B42] Uetz P (2020) The Reptile Database. http://www.reptile-database.org [Accessed on: 2020-11-5]

[B43] Ulber T (1993) Bemerkungen über cyrtodactyline Geckos aus Thailand nebst Beschreibungen von zwei neuen Arten (Reptilia: Gekkonidae). Mitteilungen aus dem Zoologischen Museum in Berlin 69: 187–200. 10.1002/mmnz.19930690202

[B44] Wilcox TP Zwickl DJ Heath TA Hillis DM (2002) Phylogenetic relationships of the dwarf boas and a comparison of Bayesian and bootstrap measures of phylogenetic support. Molecular Phylogenetics and Evolution 25: 361–371. 10.1016/S1055-7903(02)00244-0 12414316

